# Effect of Chemical Modification on Molecular Ordering in Polydiketopyrrolopyrrole Copolymers: From Liquid Crystalline to Crystalline

**DOI:** 10.1021/acs.macromol.4c00264

**Published:** 2024-05-29

**Authors:** Robert T. Kahl, Andreas Erhardt, Gert Krauss, Ferdinand Seibold, Oleksandr Dolynchuk, Mukundan Thelakkat, Thomas Thurn-Albrecht

**Affiliations:** † Experimental Polymer Physics, Martin Luther University Halle-Wittenberg, Von-Danckelmann-Platz 3, 06120 Halle, Germany; ‡ Applied Functional Polymers, University of Bayreuth, Universitätsstr. 30, 95440 Bayreuth, Germany

## Abstract

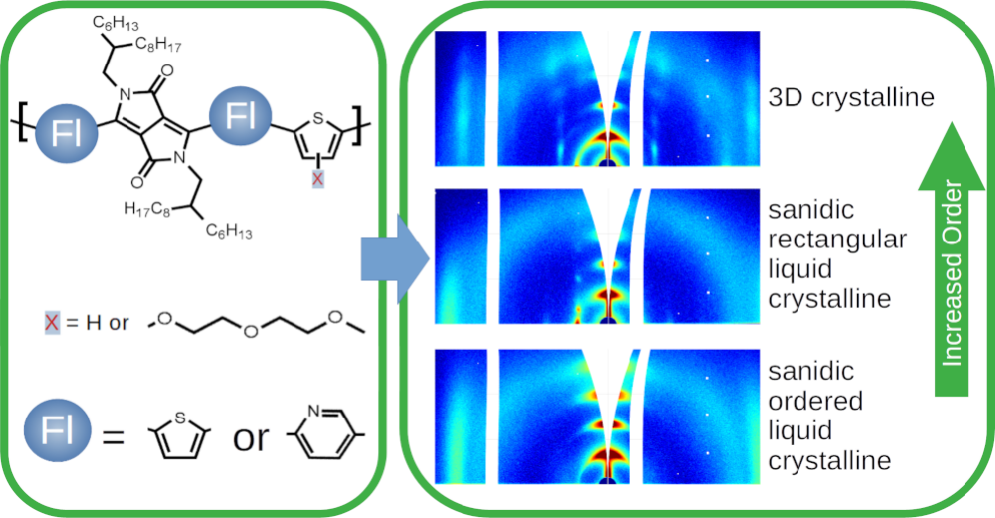

The chemical architecture of conjugated polymers is often designed by contemplating and understanding the consequences of structural changes on electronic properties at the molecular level. However, even minor changes to the chemical structure of a polymer can significantly influence the packing arrangement, which also influences the electronic properties of the bulk material. Here, we investigate the molecular arrangement in the ordered state at room temperature of a series of three different polydiketopyrrolopyrroles (PDPPs) in bulk and oriented thin films in detail by wide-angle X-ray scattering and by atomic force microscopy. The changes in the chemical structure of the investigated PDPPs, namely, an additional side chain or a different flanking unit, lead to an increase in long-range order and thereby to a change in the phase state from sanidic ordered via sanidic rectangular or oblique to crystalline.

## Introduction

Semicrystalline π-conjugated polymers have been the subject of intensive research over the past decades. They feature a unique combination of properties such as semiconductivity, light weight, mechanical flexibility, and solution processability, which makes them interesting for many different applications. For example, semiconducting polymers are used as active material in organic field effect transistors (OFETs), organic light-emitting diodes (OLEDs), organic photovoltaics (OPV), and organic electrochemical transistors (OECTs).^1−4^ For all these different applications, the mobility of charge carriers within the semiconducting polymer material and its conductivity have a significant impact on the efficiency of the final device.^5,6^ One major aspect determining the charge carrier mobility of a semiconducting polymer is the intermolecular ordering of the polymer chains. In semicrystalline conjugated polymers, the crystalline regions and their interconnectivity (tie chains) mostly determine the charge transport.^7−9^ Therefore, typically a high crystallinity is favorable for a high charge carrier mobility as this reduces the volume of the disordered amorphous regions and thereby reduces the amount of necessary tie chains for good interconnectivity of the crystalline regions.

Not only is the sheer volume fraction of crystalline regions important for the material properties, but also the molecular arrangement of the polymer chains within the crystalline regions, i.e., the crystal structure, can influence the material properties. For semiconducting polymers featuring polymorphism, it was demonstrated that even for the same polymer the charge carrier mobility can vary between different polymorphs.^10,11^ The crystal structure can also influence the optical properties of semiconducting polymers,^12,13^ which can be decisive for polymers used as active material in OPV devices.

One group of semiconducting polymers that was extensively investigated within recent years is diketopyrrolopyrrole (DPP)-based copolymers. The typical chemical structure of PDPP copolymers consists of a DPP core with attached side chains and flanking units copolymerized with a comonomer.^14^ On the one hand, the interest in PDPP is motivated by their high charge carrier mobility, which for several PDPPs has been reported to be greater than 10 cm^2^ V^–1^ s^–1^.^15,16^ On the other hand, the interest is motivated by their chemical versatility, which is based on the modularity of their chemical structure, allowing the fine-tuning of their properties according to the desired application by modifying flanking units, side chains, or comonomers. For example, it is possible to introduce chemical modifications such that the resulting PDPPs are either p-type, n-type, or ambipolar semiconductors.^14,17−20^ By replacing the alkyl side chains by ethylene glycol (EG) side chains, it is possible to synthesize PDPPs that are mixed ion–electron conductors, which can be used in bioelectronic devices, such as OECT.^21^ Due to these promising properties, a vast variety of different PDPPs have been synthesized and investigated for their applications over the past few years.^22^ However, the crystal structure of the newly synthesized PDPPs and DPP-based copolymers and the effect of the introduced chemical modifications on the crystal lattice were investigated only in a few cases.^23−26^ Especially structural evolution after crystallization from the melt is rarely considered. Different crystal orientations in thin films have been reported,^14,27^ but a detailed analysis of the type of crystal lattice or liquid crystalline structure is not available. In fact, as often for board-like polymers sanidic liquid crystalline phases are observed in experiments and simulations,^28−30^ but a detailed experimental study of the molecular ordering in these mesophases is rare. On the other hand, it was shown for several semiconducting polymers that the crystal structure can be sensitive to slight chemical modifications, which in turn can have significant consequences for the macroscopic material properties.^31,32^

Here, we investigate the influence of chemical modifications on molecular ordering in a series of three exemplary PDPPs: PDPP[T]_2_-T, PDPP[T]_2_-T_*DEG*_, and PDPP[Py]_2_-T. PDPP[T]_2_-T with two thiophene flanking units and a thiophene comonomer is a commercially available PDPP and serves as a reference sample. PDPP[T]_2_-T_*DEG*_ and PDPP[Py]_2_-T both feature one chemical modification compared to PDPP[T]_2_-T. In PDPP[T]_2_-T_*DEG*_ an additional diethylene glycol (DEG) side chain was attached to the thiophene comonomer. By addition of side chains, the polarity, solubility, and ion compatibility of the polymer can be influenced, dependent on the chemical structure of the added substituents. If a sufficient amount of the side chains is polar, the resulting polymer shows mixed ion–electron conductivity.^21^ In PDPP[Py]_2_-T the thiophene flanking units are replaced by pyridine flanking units which changes the polymer from an electron-rich (donor) to an electron-deficient (acceptor) semiconductor.^19^ We investigated the molecular ordering of these three PDPPs in bulk and thin films by wide-angle X-ray scattering (WAXS), making use of the uniaxial orientation in thin films. The surface morphologies of the thin films are further studied with atomic force microscopy (AFM). We find that the introduced chemical modifications indeed have a significant influence on molecular ordering. PDPP[T]_2_-T and PDPP[T]_2_-T_*DEG*_ both adopt a sanidic liquid crystalline order,^33^ with an observable increase in order upon introduction of the DEG substituents. In contrast, PDPP[Py]_2_-T is crystalline with a triclinic unit cell.

## Experimental Section

### Materials

**Figure 1:**
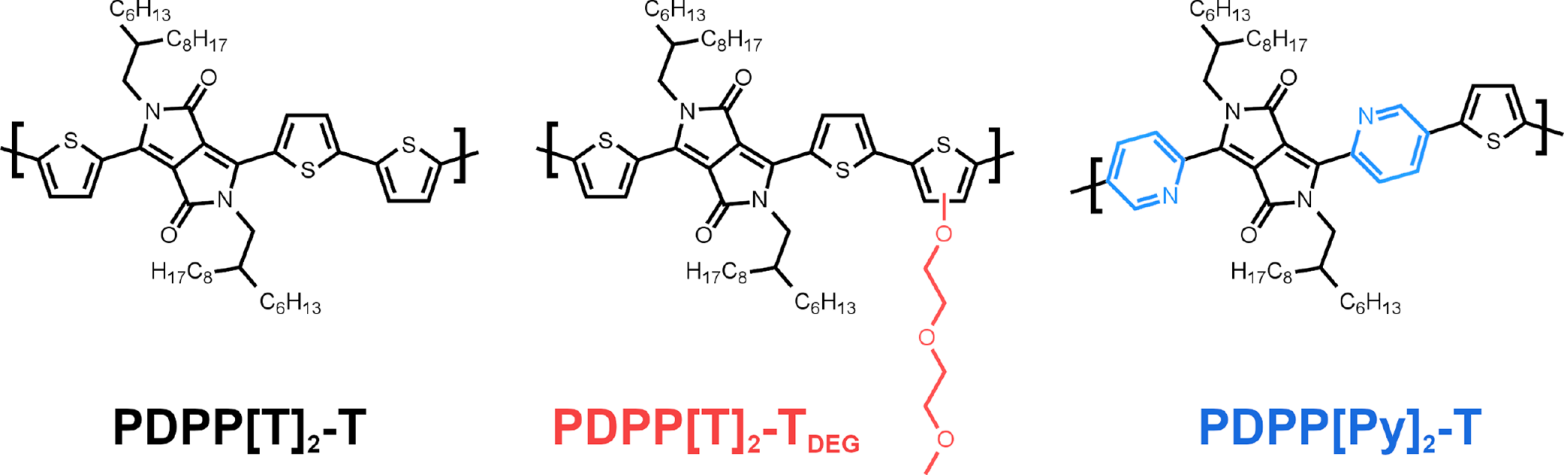
Chemical structure of the three investigated PDPPs. The changes in the chemical structure of PDPP[T]_2_-T_*DEG*_ and PDPP[Py]_2_-T with regard to PDPP[T]_2_-T are highlighted in color.

The chemical structures of the three investigated PDPPs are listed in Figure 1. PDPP[T]_2_-T is a commercially available sample purchased from Ossila, Sheffield, England, and was used as received. The synthesis of PDPP[T]_2_-T_*DEG*_ and PDPP[Py]_2_-T is described in previous publications.^19,21^ The molecular weight and the dispersity *Đ* of the investigated PDPPs were determined by gel permeation chromatography (GPC) and are given in Table 1. Chloroform was used as eluent, and polystyrene was used as calibration standard.

**Table 1:** Summary of the Polymer Properties: Molecular Weight, Dispersity, Thermal Properties, and Lamellar and π–π Spacings

polymer	*M*_n_^a^ [kDa]	*Đ*^a^	*T*_5%_^dynamic^ b [°C ]	*T*_5%_^isotherm^ c [°C ]	*T*_m_^d^ [°C ]	Δ*H*_m_^d^ [J/g]	*d*_100_^e^ [Å]	*d*_010_^e^ [Å]
PDPP[T]_2_-T	45.4	2.94	408.4	330	289.1	25.0	19.39	3.91
PDPP[T]_2_-T_*DEG*_	17.6	1.94	371.3	300	244.3	11.5	18.98	3.70
PDPP[Py]_2_-T	43.5	1.8	379.1	200	284.7	6.9	18.81	3.88

a Determined by GPC with polystyrene calibration and THF as eluent.

b Determined by dynamic TGA.

c Determined by stepwise isothermal TGA.

d Determined by DSC.

e Determined by WAXS as discussed below.

### Thermogravimetric Analysis (TGA)

TGA thermograms were recorded using a Mettler Toledo TGA/DSC 3+ under a N_2_ atmosphere. Dynamic measurements were performed in a temperature range from 30 to 700 °C at a constant heating rate of 10 K min^–1^. For the isothermal measurements, isothermal annealing steps were conducted for 1 h in steps of 10 K in the intervals 130 to 500 °C or 190 to 500 °C. The heating rate between isothermal steps was 10 K min^–1^. Specimens with a weight of approximately 7 mg were filled into Al_2_O_3_ crucibles with a volume of 70 μL.

### Differential Scanning Calorimetry (DSC)

A PerkinElmer DSC 8000 was used with a heating/cooling rate of 10 K min^–1^ under a N_2_ atmosphere. Specimens with a weight of approximately 3 to 5 mg were filled into aluminum sample pans. After the samples were measured for three consecutive heating and cooling runs, the sample weight was remeasured to observe any potential weight loss. The PDPP[T]_2_-T and PDPP[T]_2_-T_*DEG*_ samples showed no significant weight loss (<0.1%). For the PDPP[Py]_2_-T sample a weight loss of ≈6.4% was observed.

### X-ray Scattering

WAXS and grazing-incidence wide-angle X-ray scattering (GIWAXS) patterns were measured with a laboratory setup Retro-F from SAXSLAB (Copenhagen, Denmark) equipped with a microfocus X-ray source from AXO (Dresden, Germany) and an AXO multilayer X-ray optics (ASTIX) as a monochromator for Cu Kα radiation (λ = 0.15418 nm). A PILATUS R 300K detector from DECTRIS (Daettwil, Switzerland) was used to record 2D scattering patterns. WAXS measurements on bulk samples were performed in transmission geometry and thin film measurements in reflection geometry. All measurements were performed under vacuum at room temperature with a sample–detector distance of around 90 mm. For GIWAXS measurements an angle of incidence α_i_ = 0.18° was chosen, in between the critical angle of the substrate (α_c_(Si) °, α_c_(SiO_2_) °), and the polymers (α_c_(PDPPs) °). Additionally, measurements at an angle of incidence of α_i_ = 10° were performed to access reflections at a larger *q*-value positioned close to the *q*_*z*_-axis.

### Atomic Force Microscopy (AFM)

AFM measurements were performed with a Bruker MultiMode 8 AFM with a Nanoscope V controller in peak force tapping mode, which in our experience typically gives better images of very soft materials than common tapping mode. The ScanAsyst-Fluid+ cantilevers (*f*_0_ = 150 kHz, *k* = 0.7 N m^–1^) used for the measurements were purchased from Bruker. The cantilever was operated at an excitation frequency of 2 kHz. The obtained AFM images were processed and analyzed using the open-source software Gwyddion.^34^

### Thin Film Preparation

The polymer powder was dissolved in chloroform to obtain solutions with a concentration of 1 wt %. The solutions were spin-coated onto silicon substrates with a spinning speed of 2000 rpm and a spinning duration of 60 s, resulting in a film thickness of about 100 nm as determined by AFM. The silicon substrates were cut from a silicon wafer with a naturally oxidized silicon oxide layer of 2–3 nm. The resulting substrates had a size of approximately 1 cm × 1 cm. The substrates were cleaned in sulfuric acid for 30 min. After the substrates were rinsed with distilled water, the substrates were heated to 160 °C in a vacuum oven and kept at this temperature for 1 h for drying. Directly before spin-coating, the substrates were cleaned with a CO_2_-snowjet. The spin-coated films were ordered during cooling from the melt (PDPP[T]_2_-T, PDPP[T]_2_-T_*DEG*_) or annealed (PDPP[Py]_2_-T) on a Linkam hot stage under vacuum.

### Scattering from Uniaxially Aligned Polycrystalline Thin Films

**Figure 2:**
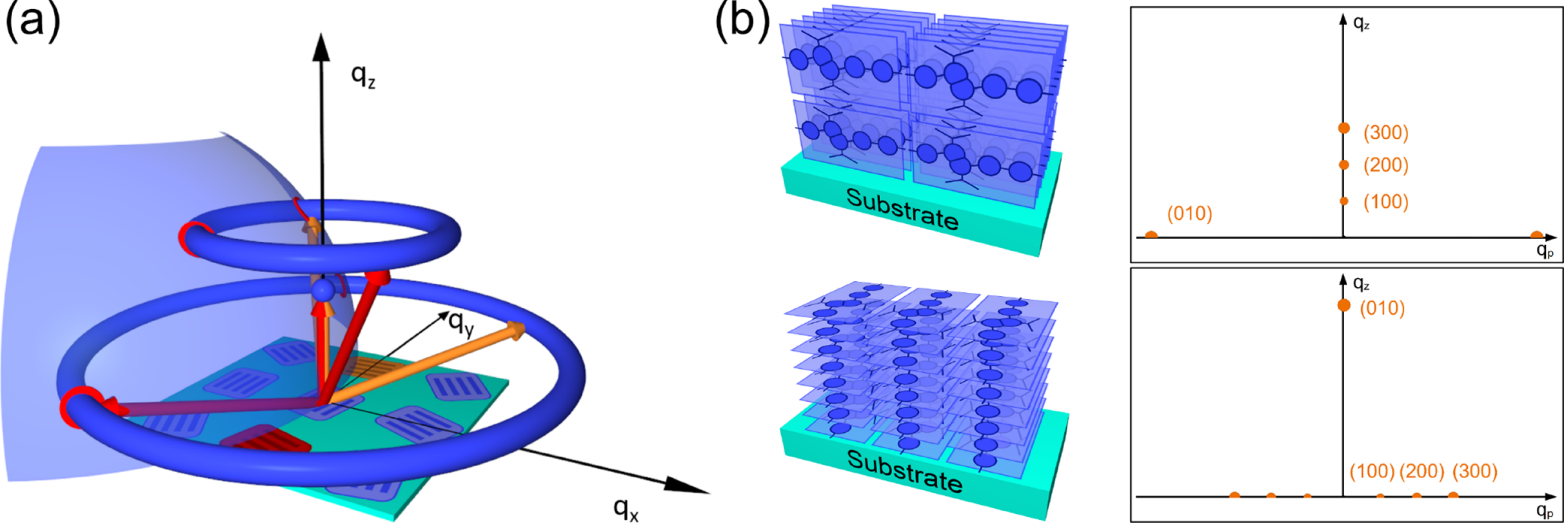
(a) Sketch of the intensity distribution in reciprocal space of polycrystalline thin film samples with crystals oriented with respect to the surface normal (*q*_*z*_) but random orientation within the plane. Because of the superposition of the contributions from the individual crystals, the intensity distribution shows rotational symmetry around the *q*_*z*_-axis, resulting in ring-like distributions of all reflections not directly on the *q*_*z*_-axis. Red and orange arrows indicate positions of same reflection for two differently oriented crystals. A scattering signal on the detector is observed at positions where the rings intersect (red circles) the Ewald sphere (light blue sphere). (b) Sketch of edge-on and face-on oriented crystals together with the corresponding scattering patterns in reciprocal space.

Often the large surface-to-volume ratio in crystalline thin films leads to strong orientation effects, and a specific lattice plane preferentially aligns parallel to the substrate, while the sample remains isotropic within the plane of the substrate. Figure 2a shows a sketch of the corresponding intensity distribution in reciprocal space. Reciprocal lattice points, i.e., Bragg reflections from the family of oriented lattice planes, lie on the *q*_*z*_-axis; other Bragg reflections are distributed on circular rings around the *q*_*z*_-axis (cf. Figure 2a). This uniaxial orientation distribution is similar to that of a drawn fiber and can be used to advantage for structural analysis in scattering experiments. The advantage of measurements on thin films compared to drawn fibers is that already a small amount of material (1 to 3 mg, depending on the desired film thickness) is sufficient to prepare multiple thin film samples by spin-coating. However, in detail the alignment commonly found in thin films differs from the one found in fibers. The direction of alignment in thin films is the surface normal, whereas in drawn fibers it is the drawing direction. In fibers the polymer backbones typically align parallel to the drawing direction. This orientation would correspond to a so-called chain-on orientation in thin films where the chains are arranged parallel to the surface normal. The chain-on orientation though is rather seldom observed; typically the polymer chains are preferentially aligned parallel to the substrate and therefore perpendicular to the orientation axis (surface normal). For semiconducting polymers composed of board-like monomers with attached side chains, this would correspond to edge-on alignment, i.e., side chains parallel to the surface normal, or face-on alignment, i.e., π–π stacking direction parallel to the surface normal, as sketched in Figure 2b on the left.

Scattering experiments on thin film samples are typically performed in grazing incidence geometry (GIWAX: grazing incidence wide-angle X-ray scattering), where the Ewald sphere cuts the *q*_*z*_-axis at a small distance from the origin of reciprocal space as sketched in Figure 2a (Ewald sphere in light blue). Intensity from the reflections not lying on the *q*_*z*_-axis is observed wherever the corresponding rings intersect with the Ewald sphere. With the *q*_*z*_-axis chosen perpendicular to the film surface and the *q*_*x*_- and *q*_*y*_-axis lying parallel to the film surface, the scattering pattern observed on a 2D detector can be converted into a reciprocal space map with the following formulas, which take into account refraction effects.^35^
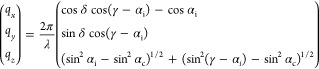
1Here δ is the in-plane scattering angle, γ is the out-of-plane scattering angle, α_i_ is the incident angle of the primary beam, and α_c_ is the critical angle of the polymer. Taking into account the cylindrical symmetry of the uniaxial orientation distribution, the 2D intensity distribution in reciprocal space is plotted as a function of  and *q*_*z*_. The accessible region of the reciprocal space depends on α_*i*_.

## Results and Discussion

### Chemical Stability and Thermal Behavior

#### TGA

**Figure 3:**
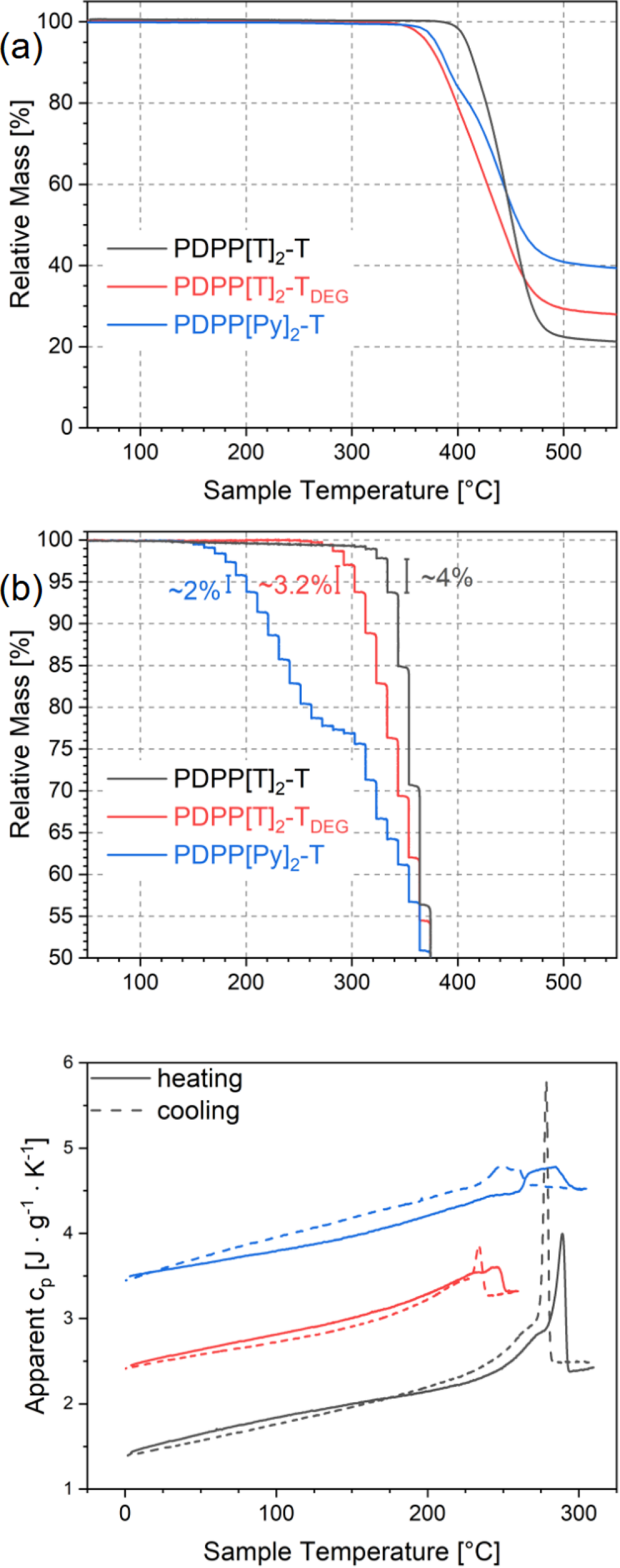
(a) Dynamic and (b) isothermal TGA and (c) DSC measurements of PDPP[T]_2_-T (black), PDPP[T]_2_-T_*DEG*_ (red), and PDPP[Py]_2_-T (blue). The scale bars in (b) indicate the relative mass loss during the annealing step during which the relative sample mass fell below 95%. The upper two curves in (c) are shifted vertically by 1 J g^–1^ K^–1^ relative to one another for better visibility.

All samples showed a high thermal stability up to about 370 °C (5% loss) in common dynamic thermogravimetric analysis (TGA) under a N_2_ atmosphere with a heating rate of 10 K min^–1^ as shown in Figure 3. Nevertheless, as we observed indications of sample degradation in repeated DSC measurements for PDPP[Py]_2_-T as dicussed below (cf. Figure S1), we performed additional isothermal TGA measurements with a stepwise heating program with steps of 10 K and an annealing time of 1 h at each step. These measurements give relevant information for further sample treatment as annealing and crystallization from the melt typically keep the sample at elevated temperatures for longer times than modeled by a dynamic TGA measurement. Because of the extended duration of the isothermal segments, however, the results shown in Figure 3a give an upper limit of the material deterioration during the experiments described below. While PDPP[T]_2_-T and PDPP[T]_2_-T_*DEG*_ are still thermally relatively stable and a total mass loss of 5 wt % is only reached at 330 and 300 °C, respectively, PDPP[Py]_2_-T starts to degrade comparably early, already reaching a total mass loss of 5 wt % at only 200 °C. However, as the weight loss of the pyridyl-flanked PDPP in dynamic TGA is not substantial before the degradation onset of the other materials, deterioration of a distinct structural site seems unlikely, and we cannot give a concrete explanation for the lower onset for weight loss of PDPP[Py]_2_-T revealed via isothermal TGA investigations.

#### DSC

The melting and crystallization behavior of the polymers was investigated by differential scanning calorimetry (DSC) with a heating/cooling rate of 10 K min^–1^. Figure 3b shows the resulting measurements. For PDPP[T]_2_-T and PDPP[T]_2_-T_*DEG*_ the first cooling and second heating runs are shown. For PDPP[Py]_2_-T the first cooling and the first heating run after a preceding annealing program are shown instead, as the second and third heating runs are obviously affected by degradation (cf. Figure S1). The annealing program serves to crystallize the sample and is the same as that used for the WAXS measurements. It is shown in Figure S2. While PDPP[T]_2_-T and PDPP[Py]_2_-T have similar melting temperatures of 289.1 and 284.7 °C, respectively, the melting temperature of PDPP[T]_2_-T_*DEG*_ is only 244.3 °C. A similar reduction of the melting temperature was also observed for polythiophenes, where replacing the hexyl side chains of poly(3-hexylthiophene) (P3HT) by DEG side chains also resulted in a drastic reduction of the melting temperature.^36^ Furthermore, it is indeed apparent that the melting temperature of PDPP[Py]_2_-T lies substantially above its long time degradation temperature as observed in the isothermal TGA. For this reason, the PDPP[Py]_2_-T samples for DSC, WAXS, and GIWAXS measurements were only annealed at an intermediate temperature and not ordered during cooling from the melt to minimize the degradation during sample preparation. The results of the DSC and TGA measurements are summarized in Table 1.

### Structure Determination

#### WAXS on Bulk Samples

**Figure 4:**
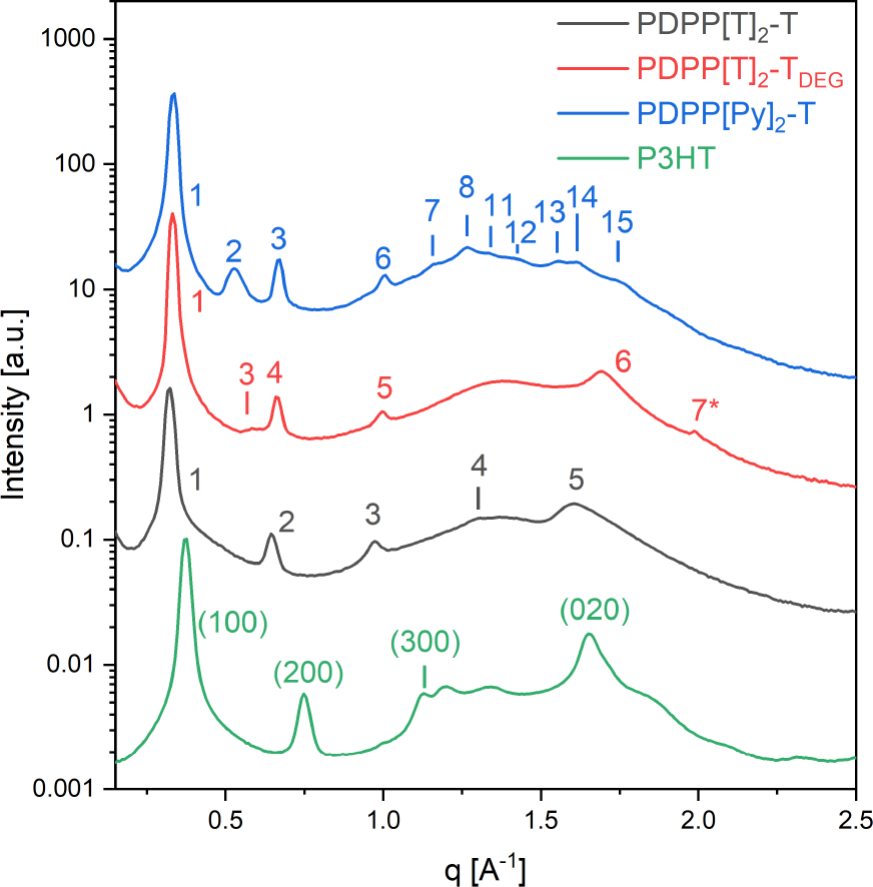
Powder WAXS patterns of PDPP[T]_2_-T (black) and PDPP[T]_2_-T_*DEG*_ (red) after crystallization by cooling from the melt and PDPP[Py]_2_-T (blue) after annealing at 260 °C. A powder WAXS measurement of P3HT (green) crystallized from the melt is shown for comparison to emphasize similarities of molecular ordering in semiconducting polymers consisting of a rigid conjugated backbone with attached flexible side chains: regular stacking of backbones giving rise to (0*k*0) reflections and regular layers formed by stacked backbones separated by layers of side chains giving rise to (*h*00) reflections. Curves are shifted on the *y*-axis for better visibility. All measurements were performed at room temperature.

Figure 4 shows the WAXS patterns at room temperature of the three investigated PDPPs in comparison to that of P3HT. The powder scattering patterns were measured after the following thermal treatment of the samples. PDPP[T]_2_-T, PDPP[T]_2_-T_*DEG*_, and P3HT were ordered during cooling from the melt; PDPP[Py]_2_-T was heated stepwise with a final annealing step at 260 °C only in order to minimize degradation (cf. Figure S2 for the precise temperature program). (A sample of PDPP[Py]_2_-T cooled from the melt measured for comparison showed only poor ordering, presumably due to degradation during elongated time in the melt state.) The observed scattering peaks are numbered according to their *q*-position in ascending order, including the peaks from the GIWAXS measurements shown further below.

The scattering patterns of the PDPPs show similarities to the scattering pattern of P3HT: a series of equidistant peaks in the *q*-range from 0.3  to 1.2 Å^–1^ and at least one additional peak in the *q* range from 1.5 to 1.8 Å^–1^. The general chain architecture of the investigated PDPPs is similar to that of P3HT: a rigid board-like backbone with flexible side chains. Therefore, it is likely that certain motifs of molecular arrangement observed in P3HT that are related to this architecture can also be found in the investigated PDPPs. Namely, the periodic π–π stacking of the conjugated backbones, giving rise to the (0*k*0) reflection, and the formation of a regularly layered structure formed by a separation of stacked backbones and side chains, giving rise to a series of equidistant (*h*00) reflections. In P3HT certain reflections can only be explained by choosing a unit cell containing two monomers in the π–π stacking direction, resulting in an indexing of the π–π stacking peak as the (020) reflection instead of the (010) reflection.^37^ In the investigated PDPPs all observed diffraction peaks can be indexed without having to assume more than one monomer per unit cell. Accordingly, we index the π–π stacking peak as a (010) reflection. Based on the assumption of similar motifs in the molecular arrangement of board-like chain molecules, it is possible to index some of the peaks in the scattering patterns of the investigated PDPPs as (*h*00) and (010) reflections. The *d*_100_ and *d*_010_ spacings determined from the WAXS measurements are given in Table 1. While the π–π stacking distances of the investigated PDPPs are quite similar to that of P3HT (*d*_020,P3HT_ = 3.81 Å), the *d*_100_ spacings of the PDPPs are significantly larger than that found in P3HT (*d*_100,P3HT_ = 16.68 Å), which is to be expected based on the longer side chains in the PDPPs. The remaining peaks cannot be indexed based on the observed similarity of the scattering patterns to that of P3HT alone. Here measurements from oriented samples are necessary, as the orientation allows one to obtain more information about the position of the observed reflections in reciprocal space. Therefore, we conducted GIWAXS measurements on thin film samples taking advantage of the alignment effects in thin films, as discussed in the Experimental Section.

#### GIWAXS on Thin Films

**Figure 5:**
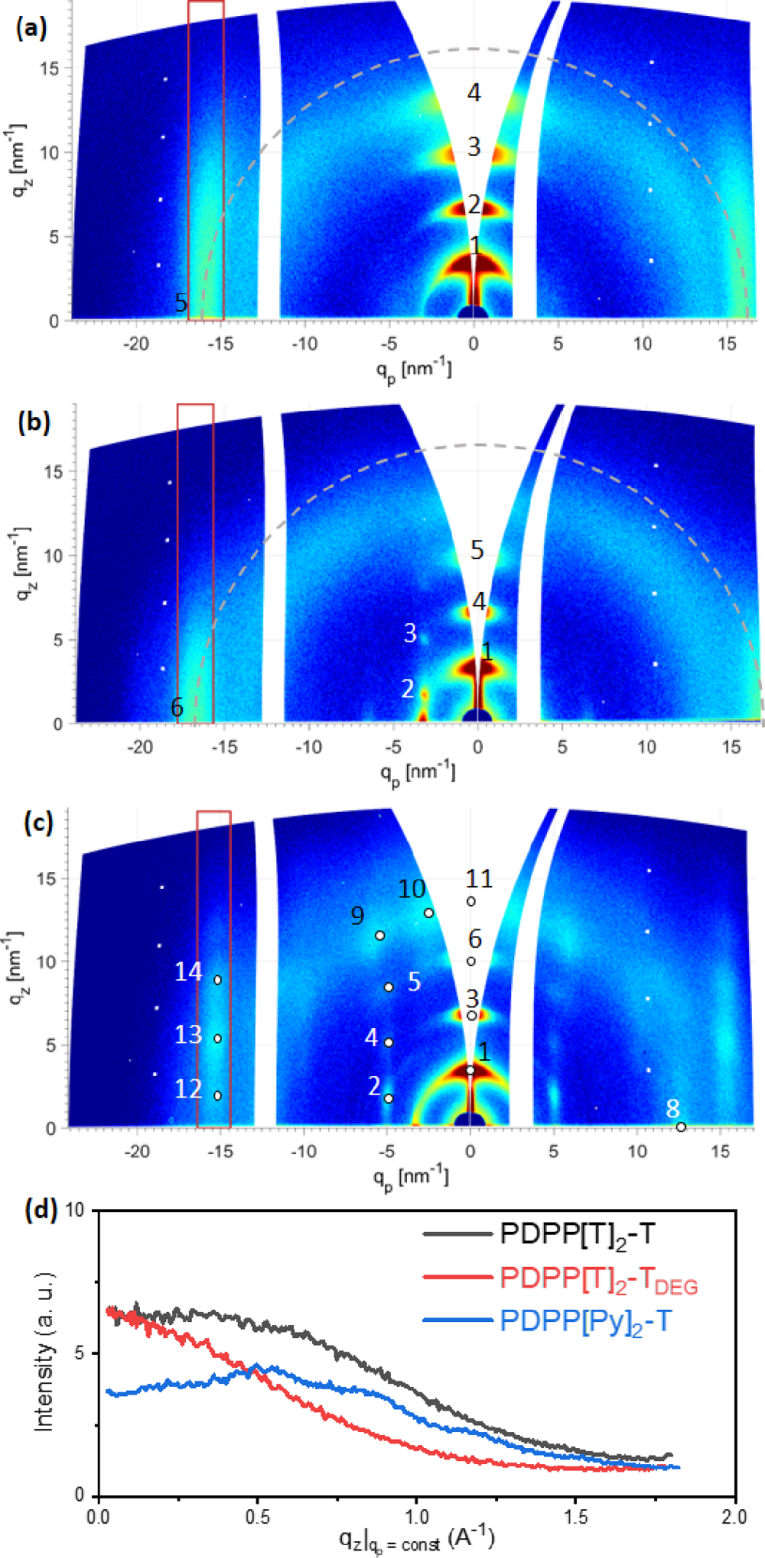
GIWAXS measurements on thin films of (a) PDPP[T]_2_-T and (b)PDPP[T]_2_-T_*DEG*_ after cooling from melt and (c) PDPP[Py]_2_-T after the annealing procedure described in the text. All measurements were performed at room temperature and at an angle of incidence of 0.18°. (d) Intensity profiles in the *q*_*z*_ direction of the π–π stacking peaks of the three polymers. Red rectangles in (a–c) indicate the integration area used to extract the intensity profiles plotted in (d). The integration range in (a–c) was similar but shifted in *q*_p_ according to the position of the π–π stacking peaks. Gray dashed circles in (a) and (b) show the expected intensity distribution of peaks 5 and 6, respectively, for completely randomly oriented crystals.

Figure 5a–c shows the results of GIWAXS measurements on thin film samples of PDPP[T]_2_-T, PDPP[T]_2_-T_*DEG*_, and PDPP[Py]_2_-T. The thin films of PDPP[T]_2_-T and PDPP[T]_2_-T_*DEG*_ were ordered during cooling from the melt. PDPP[Py]_2_-T instead was annealed at an elevated temperature close to the melting temperature in order to avoid degradation in the melt state; the precise treatment together with the corresponding TGA measurement is shown in Figure S2. In this way, a well-ordered thin film sample with strong edge-on orientation could be prepared. More detailed information is given in Figures S3 and S4.

All three GIWAXS patterns show a series of (*h*00) reflections on the meridian of high intensity. Because of the splitting along the *q*_*z*_-axis in the converted detector images, only the wings of the higher orders (*h*00) reflections are visible. The presence of the (300) and (400) reflections was therefore directly verified by measurements at an angle of incidence of α_i_ ≈ 10° (cf. Figure S5). The contribution of the (*h*00) reflections on the equator is, on the other hand, of much lower intensity. From these strongly oriented (*h*00) reflections it is obvious that all three PDPPs show a strong preferential edge-on orientation. A similar preference for edge-on orientation in films of PDPP[T]_2_-T and PDPP[Py]_2_-T annealed at lower temperatures was also observed by Mueller and co-workers.^14^ This preference for edge-on orientation is likely caused by the alkyl and EG side chains, which are known to promote edge-on orientation at the interface to vacuum.^35,36,38^ Strikingly, the (010) peaks visible in the GIWAXS pattern of PDPP[T]_2_-T and PDPP[T]_2_-T_*DEG*_ (peaks 5 and 6, respectively) are rather broad streaks perpendicular to the equator instead of sharp and localized peaks. The scattering pattern for PDPP[Py]_2_-T shows an additional intensity variation along the *q*_*z*_ directions (peaks 12–14). The corresponding intensity profiles along the *q*_*z*_ direction obtained by integrating the intensity in the *q*_p_ direction are shown in Figure 5d. The range of integration in the *q*_p_ direction was chosen for each sample individually according to the different positions and widths of the (010) reflections as indicated by the red rectangles in Figure 5a–c. Based on a closer analysis of the scattering patterns in Figure 5, we discuss below the type of molecular order for the three different samples.

#### Molecular Ordering of PDPP[T]_2_-T

PDPP[T]_2_-T shows the most simple scattering pattern. Based on the arguments given above, it is straightforward to index all reflections visible in the WAXS and GIWAXS patterns; peaks 1–4 are (*h*00) reflections, and peak 5 is the (010) reflection. A list of all peak positions determined from WAXS and GIWAXS measurements is given in Table S1. As no additional peaks are visible in either the WAXS or GIWAXS measurements, there is no evidence for a periodic ordering in the backbone direction and no evidence for any coupling between the ordering in the (100) and (010) directions. This result is in keeping with transmission WAXS measurements on free-standing thin films of PDPP[T]_2_-T reported in the literature, where no reflections with mixed indices were observed and an additional (001) reflection was observed only upon applying a certain strain to the sample.^39^

**Figure 6:**
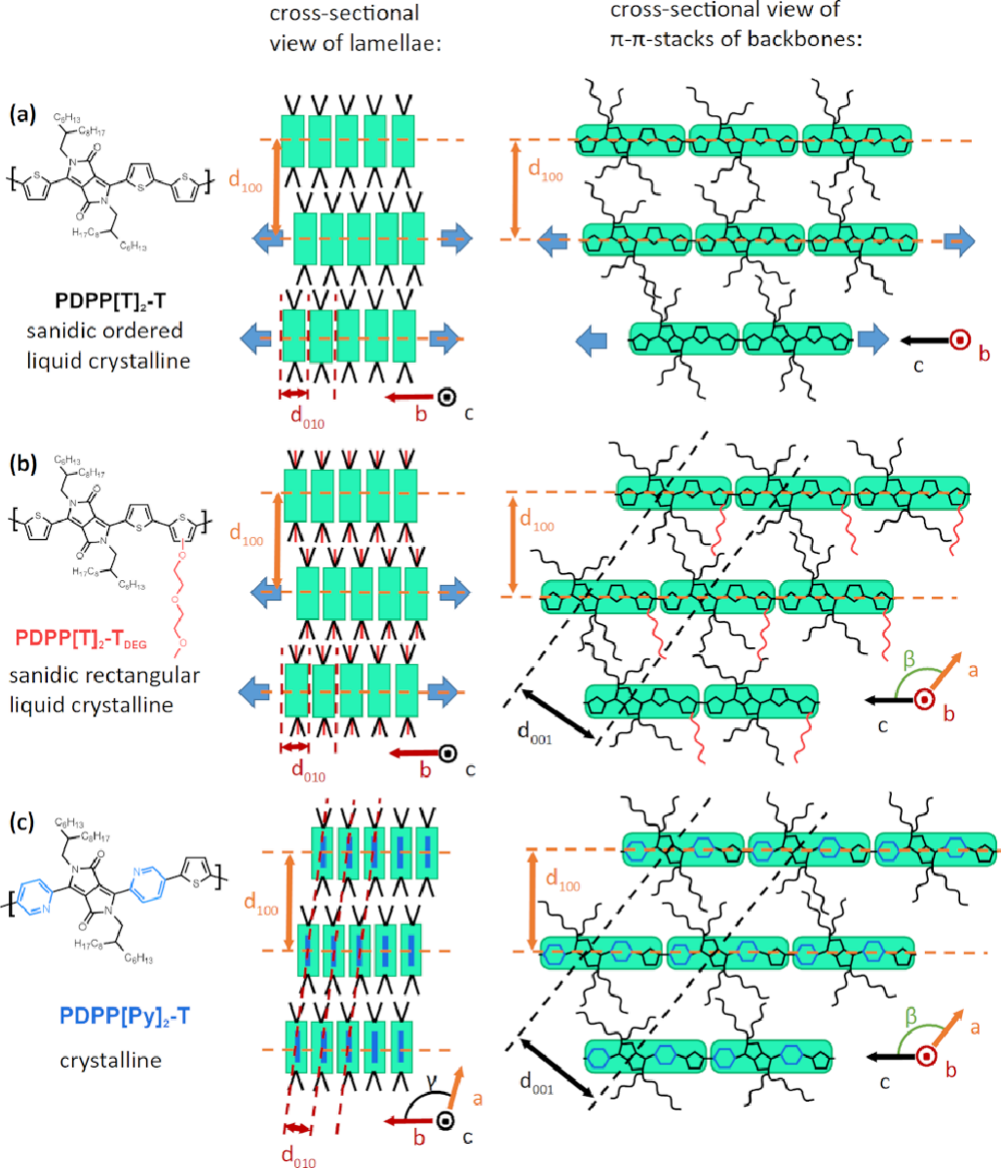
Sketches of suggested molecular arrangements in PDPP[T]_2_-T (top), PDPP[T]_2_-T_*DEG*_ (middle), and PDPP[Py]_2_-T (bottom) based on the observed scattering patterns. In PDPP[T]_2_-T neighboring stacked backbones are shifted randomly against one another in the *b* and *c* directions as indicated by the thick blue arrows. In PDPP[T]_2_-T_*DEG*_ neighboring stacks are randomly shifted in the *b* direction (indicated by the blue arrows) but registered in the *c* direction. In PDPP[Py]_2_-T neighboring stacks are registered in *b* and *c* directions.

The streak-like intensity distribution of the (010) reflection shows a clear deviation from the circular shape, as it would be expected, if it were caused by a broad orientational distribution only (cf. Figure 5a). Therefore, this broadening of the (010) reflections is at least partially caused by a limited correlation in the *q*_*z*_ direction, indicating that neighboring stacks of π–π stacked molecules separated by layers of side chains are randomly shifted against one another as sketched in Figure 6a.

Based on these results, the order displayed by PDPP[T]_2_-T is of liquid crystalline nature only and can be identified as a sanidic ordered structure Σ_o_, following the classification introduced by Ebert et al. for the liquid crystalline phases of board-like molecules.^33^ Different from the cases discussed below, the order in different directions is uncorrelated, as there are no reflections with mixed indices. An illustration of the molecular arrangement in the *b*–*c* plane is given in Figure S6.

#### Molecular Ordering of PDPP[T]_2_-T_*DEG*_

Based on the comparison with PDPP[T]_2_-T, most of the reflections in the WAXS and GIWAXS patterns of PDPP[T]_2_-T_*DEG*_ can be indexed easily. Peaks 1, 4, and 5 can be identified as (*h*00) reflections and peak 6 as the (010) reflection. Compared with the other visible peaks, peak 7* in the WAXS pattern stands out. On the one hand it is unusually sharp, and on the other hand it does not disappear upon heating the sample above the melting temperature. Furthermore, it is not observed in the GIWAXS measurements. From these observations it is clear that peak 7* is not caused by scattering from the sample but likely from some kind of crystalline impurity.

In the GIWAXS measurement (cf. Figure 5b), an additional peak 2 is visible, just above the (100) reflection located on the equator coming from the face-on oriented crystals. This peak 2 could not be seen in the WAXS scattering pattern of the powder sample as its  position is quite close to that of the much more intense (100) reflection. Since the sample is mostly edge-on oriented, peak 2 must belong to a series of reflections with either *k* or *l* ≠ 0. Given the large *q*_p_ value of the (010) reflection, peak 2 has to contain an *l* ≠ 0. We therefore assume that peak 2 is the (001) reflection, as this is the simplest assumption. Following from this indexation, peak 3 can be identified as a (101) reflection. The angles β* ≈ 61.8° (between (100) and (001) reflections) and γ* ≈ 90° (between (100) and (010) reflections) can be directly taken from the GIWAXS measurement. A list of all peak positions determined from WAXS and GIWAXS measurements is given in Table S2.

Although the (010) peak of PDPP[T]_2_-T_*DEG*_ is not as broad in the *q*_*z*_ direction as the (010) peak of PDPP[T]_2_-T, it is still streak-like rather than a sharp peak. Hence, while the correlation length of the (010) planes in PDPP[T]_2_-T_*DEG*_ is larger than in PDPP[T]_2_-T, it is still rather limited. However, different from this latter material, PDPP[T]_2_-T_*DEG*_ shows periodic order in the backbone direction (see illustration in Figure 6b). Furthermore, the existence of a reflection with mixed (101) indices (peak 3 in Figure 5b) proves that this order is correlated across side-chain layers. The fact that the (001) reflection has a finite *q*_*z*_ value indicates that the backbones are regularly shifted from one layer to the next. Note that a similar ordering of conjugated polymers has recently been observed in simulations.^30^ We speculate that the introduction of DEG side chains in PDPP[T]_2_-T_*DEG*_ leads to a demixing of the hydrophilic DEG side chains and the hydrophobic alkyl side chains, resulting in the observed registration between neighboring stacks of backbones. There is no direct evidence if the periodic order in backbone direction persists within one stack, but we believe it to be likely. This question is discussed in more detail in the Supporting Information (cf. Figure S6b and the related text).

Overall, this sample has a higher degree of order than the previous one. Neighboring stacks are still shifted irregularly against one another in the π–π stacking direction but registered along the chain direction as sketched in Figure 6b. Because of the fact that there is a peak with mixed indices, namely, (101), this structure is similar to the sanidic rectangular phase Σ_r_ following again the classification introduced by Ebert, although in his case the correlation is between the *b* direction and the *c* direction. However, since in our case the angle between correlated directions, namely the *a* direction and *c* direction, is not equal to 90°, a more precise name would probably be sanidic oblique.

#### Molecular Ordering of PDPP[Py]_2_-T

The scattering patterns of PDPP[Py]_2_-T show many more reflections than PDPP[T]_2_-T_*DEG*_, suggesting that this material forms a real 3-dimensional crystal. Peaks 1, 3, 6, and 11 can directly be identified as (*h*00) reflections. The (010) reflection cannot be assigned from the WAXS pattern alone, as there are multiple peaks roughly matching the expected *q* value, namely, peaks 12, 13, and 14. From the GIWAXS measurement it becomes obvious that these three peaks 12, 13, and 14 have the same *q*_p_ position and are regularly spaced in the *q*_*z*_ direction with a spacing that corresponds to *q*_100_, indicating that they form a series of (*h*10) reflections. Following similar cases in previous reports, we index the most intense reflection, i.e., peak 13, as the (010) reflection.^35,40^ Accordingly, peak 12 is the  reflection and peak 14 is the (110) reflection. Analogous to the case of PDPP[T]_2_-T_*DEG*_, peak 2 in PDPP[Py]_2_-T cannot be an (*hk*0) reflection, and we therefore assign indices (001) to it. It is obvious that peaks 2, 4, and 5 form a series of (*h*01) reflections; consequently, we assign the indices (101) and (201) to peaks 4 and 5, respectively. Based on the reflections indexed so far, it is clear that PDPP[Py]_2_-T takes on a crystalline structure indeed. All parameters of the reciprocal unit cell except for the angle α* (the angle between b* and c*) can be determined from the Bragg reflections analyzed above. To determine α*, a reflection with mixed *k* and *l* indices is required. Peak 8, which can be indexed as  as described in detail in the Supporting Information, can be used for that purpose (cf. Figures S7 and S8 and related text). The resulting parameters of the reciprocal and the real unit cell are given in Table 2. This unit cell is triclinic, which is in contrast to the orthorhombic unit cells suggested for other PDPPs in the literature, albeit without clear evidence.^23,24^ Sketches of the molecular arrangement of PDPP[Py]_2_-T in the crystalline phase are shown in the bottom row of Figure 6 and Figure S6c.

**Table 2:** Unit Cell Parameters of the Investigated PDPPs

reciprocal unit cell						
	a* [Å^–1^]	b* [Å^–1^]	c* [Å^–1^]	α* [deg]	β* [deg]	γ* [deg]
PDPP[T]_2_-T	0.324	1.605				≈ 90
PDPP[T]_2_-T_*DEG*_	0.331	1.698	0.366		61.8	≈ 90
PDPP[Py]_2_-T	0.334	1.620	0.530	45.7	70.0	71.3

real unit cell						
	*a* [Å]	*b* [Å]	*c* [Å]	α [deg]	β [deg]	γ [deg]
PDPP[Py]_2_-T	20.17	5.46	16.82	131.4	100.0	97.0

Based on this unit cell, also the remaining peak 7 from the WAXS pattern can be indexed as the  reflection. With this, all peaks observed in the WAXS pattern have been indexed. A list of all peak positions determined from WAXS and GIWAXS measurements together with *q* values calculated from the determined unit cell is given in Table S3. For the GIWAXS pattern, however, no (*hkl*) reflections explaining peaks 9 and 10 could be found. Peaks 9 and 10 are qualitatively different from the other peaks visible in the GIWAXS pattern, as they are less intense and relatively broad. Although the exact origin of the peaks is unclear, we therefore believe that they are caused by diffuse scattering. Apparently also for PDPP[Py]_2_-T the chemical heterogeneity along the chain, which is here caused by introduction of the pyridine rings into the backbone, goes along with an increased registration of order between different directions.

### Thin Film Morphology

**Figure 7:**
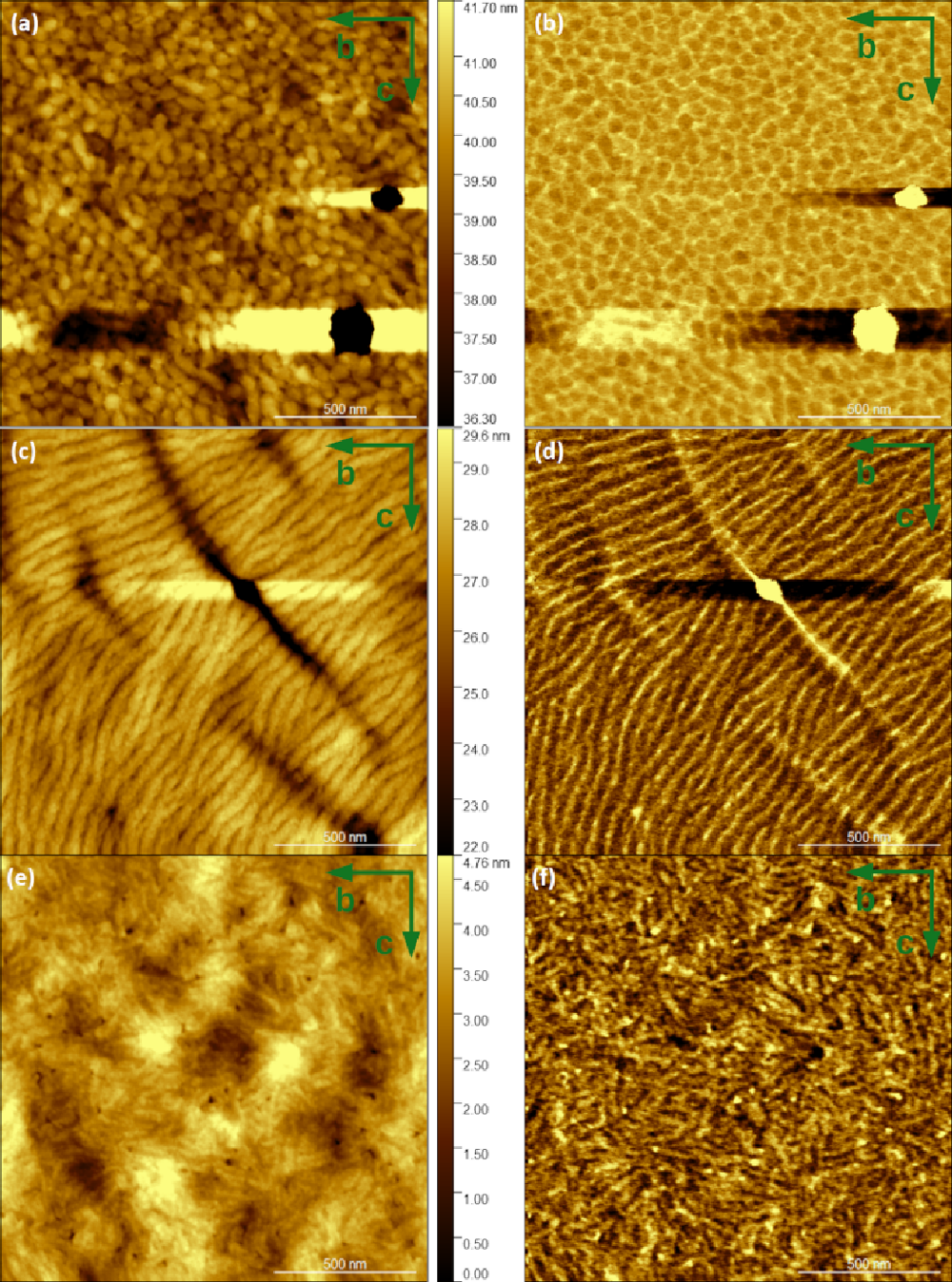
AFM height (a, c, e) and adhesion (b, d, f) images of PDPP[T]_2_-T (a, b), PDPP[T]_2_-T_*DEG*_ (c, d), and PDPP[Py]_2_-T (e, f) thin films. The isolated bright and dark spots and the neighboring horizontal streaks are artifacts from impurities on the surface. The green arrows indicate that the plane of the images corresponds to the *b*–*c* plane of the samples. Of course, the specific directions within the plane are not defined and vary across the surfaces.

The surface morphologies of thin films on silicon substrates after thermal treatment as described above were studied with AFM in peak force tapping mode. Height and adhesion images, which typically give the best signals (Figure 7), revealed significant morphological differences between the different samples. Interestingly, only the more ordered samples PDPP[T]_2_-T_*DEG*_ and PDPP[Py]_2_ show the common lamellar morphology of semicrystalline polymers (cf. Figures 7c–f), whereas the least ordered sample PDPP[T]_2_-T shows a mostly granular morphology presumably of ordered domains with a typical size of about 63 nm. Only a few domains are somewhat elongated. It seems that lamellar morphology goes along with the ordered arrangement of the chains in the *c* direction as manifested in the (001) reflection. The PDPP[T]_2_-T_*DEG*_ film (Figures 7c,d) exhibited especially clear long, stacked lamellae with an average periodicity of 56 nm. Furthermore, some cracks are visible on the surface of the PDPP[T]_2_-T_*DEG*_ film. Although their exact origin is unclear, we speculate that they were caused by internal stresses in the film due to thermal contraction upon cooling. Interestingly, the cracks all run perpendicular to the lamellae and are therefore parallel to the chain direction. As the liquid crystalline domains are likely edge-on oriented (cf. Figure 5b), the lamellae broke apart along the π–π stacking planes. Furthermore, by comparison of the images in Figure 7a–d, it is evident that the addition of DEG side chains increases the order correlation length in the *b* direction. The lamellar morphology in the PDPP[Py]_2_-T film (Figures 7e,f) consists of crystalline lamellae obviously separated by larger amorphous layers. The average long period is about 30 nm. The lamellae here are thinner, shorter, and less oriented than for the PDPP[T]_2_-T_*DEG*_ film. The differences between the latter two samples might be connected to the different molecular weight. In addition, PDPP[Py]_2_-T was only annealed below the melting temperature and not ordered during cooling from the melt.

## Conclusion

Using a combination of WAXS on isotropic bulk samples and GIWAXS on oriented thin films, we investigated the molecular ordering of three different exemplary PDPPs: PDPP[T]_2_-T containing two thiophene flanking units, PDPP[T]_2_-T_*DEG*_ with an additional DEG-side chain attached to the comonomer, and PDPP[Py]_2_-T with pyridine instead of thiophene flanking units. All samples exhibited preferred edge-on alignment on silicon substrates when annealed close to the melting temperature or ordered during cooling from the melt, which enabled us to assign all peaks in the scattering patterns. While all three PDPPs showed regular π–π stacking as well as a regular spacing in the side-chain direction, the chemical differences led to different types of sanidic order as is generally typical for board-like molecules. We observed increasing order going from PDPP[T]_2_-T arranging in a sanidic ordered liquid-crystalline phase to PDPP[T]_2_-T_*DEG*_ showing a sanidic rectangular or oblique liquid-crystalline phase to PDPP[Py]_2_-T arranging in a crystalline phase with a triclinic unit cell. On a mesoscopic scale AFM experiments revealed different morphologies for the three materials that were at least for the more strongly ordered systems reminiscent of the typical lamellar morphology of semicrystalline polymers. We hypothesize that the increasing order is related to better registration of neighboring stacks of backbones induced by the chemical heterogeneities along the backbone.

## Supporting Information Available

DSC, TGA, and GIWAXS measurements and analysis (PDF)
