# Supplementary material for: Effect of Chemical Modification on Molecular Ordering in Polydiketopyrrolopyrrole Copolymers: From Liquid Crystalline to Crystalline
Source: Macromolecules. 2024 May 29;57(11):5243–52. doi: 10.1021/acs.macromol.4c00264 (PMC11173490; doi:10.1021/acs.macromol.4c00264)
Supplement: Supplementary file 1 — ma4c00264_si_001.pdf [file ma4c00264_si_001.pdf]

Supporting Information: Effect of Chemical Modification on Molecular Ordering in Polydiketopyrrolopyrrole Copolymers: From Liquid Crystalline to Crystalline

Robert T. Kahl,[†] Andreas Erhardt,[‡] Gert Krauss,[‡] Ferdinand Seibold,[‡] Oleksandr Dolynchuk,[†] Mukundan Thelakkat,[‡] and Thomas Thurn-Albrecht[*,†]

†*Experimental Polymer Physics, Martin Luther University Halle-Wittenberg, Von-Danckelmann-Platz 3, 06120 Halle, Germany*

‡*Applied Functional Polymers, University of Bayreuth, Universitätsstr. 30, 95440 Bayreuth, Germany*

E-mail: thomas.thurn-albrecht@physik.uni-halle.de

Degradation of PDPP[Py]$_2$-T during consecutive heating and cooling runs in DSC

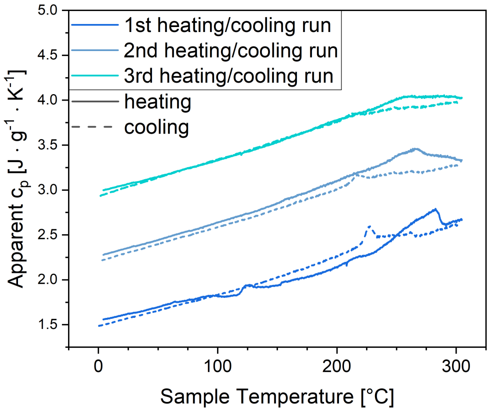

Figure S1: DSC measurements of PDPP[Py]$_2$-T from three consecutive heating and cooling runs.

To check for reproducibility several consecutive DSC measurements were performed on each sample. The same sample was subjected to three consecutive heating and cooling cycles with a heating/cooling rate of $10\,\mathrm{K\,min^{-1}}$ and a short isothermal step of 1 min at $T_{max} = 305\,°\mathrm{C}$ required by the instrument software. The result of these measurements for PDPP[Py]$_2$-T is shown in Fig. S1. There is a continuous reduction of melting and crystallization enthalpies as well as in melting and crystallization temperatures, most likely caused by sample degradation. A comparison of the sample mass before and after the DSC measurements evidenced a weight loss of 6.4 %.

Degradation during thermal treatment of PDPP[Py]$_2$-T for WAXS and GIWAXS measurements

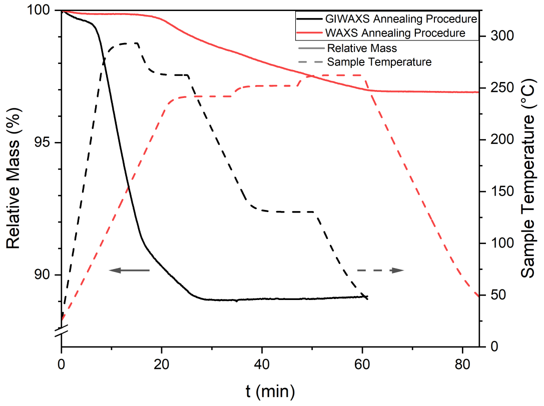

Figure S2: TGA measurement (solid line) of PDPP[Py]$_2$-T, following the same temperature program (dashed line) as used for annealing in DSC and WAXS(red) and GIWAXS(black) measurements, respectively.

Fig. S2 shows the thermal treatment used to prepare samples of PDPP[Py]$_2$-T for WAXS and GIWAXS measurements (dashed lines) in order to get well-ordered and in case of GI-WAXS additionally edge-on oriented samples. For DSC the same temperature program as for WAXS was used. To quantify the extent of degradation of PDPP[Py]$_2$-T during the chosen thermal programs TGA measurements reproducing the thermal treatment were performed (solid lines). The annealing program used for the WAXS samples resulted in a mass loss $<5\,\%$. The degradation should therefore have no significant influence on the WAXS results. The annealing program used for the GIWAXS sample resulted in a mass loss $>10\,\%$. Nevertheless, as the positions of the observed scattering peaks coincide well with the position of the peaks observed in the WAXS measurements, we can safely assume that the crystal structure was not significantly influenced by the degradation.

Thermal treatment of PDPP[Py]$_2$-T thin film samples for GIWAXS

As the onset of thermal degradation during longer time annealing of PDPP[Py]$_2$-T is below the melting temperature, a thermal treatment resulting in a balance between well ordered and oriented crystals and minimal degradation has to be found. For this purpose we performed a series of GIWAXS measurements after annealing a sample at stepwise increasing temperatures. The sample was heated to elevated temperatures between 130 °C and 240 °C in steps of 10 K. At each temperature the sample was kept for 12 minutes, then cooled to 20 °C and measured in GIWAXS. The heating/cooling rate for the first annealing step at 130 °C was 10 K min^{-1}. For all following annealing steps the heating/cooling rate was increased to 30 K min^{-1} to minimize sample degradation.

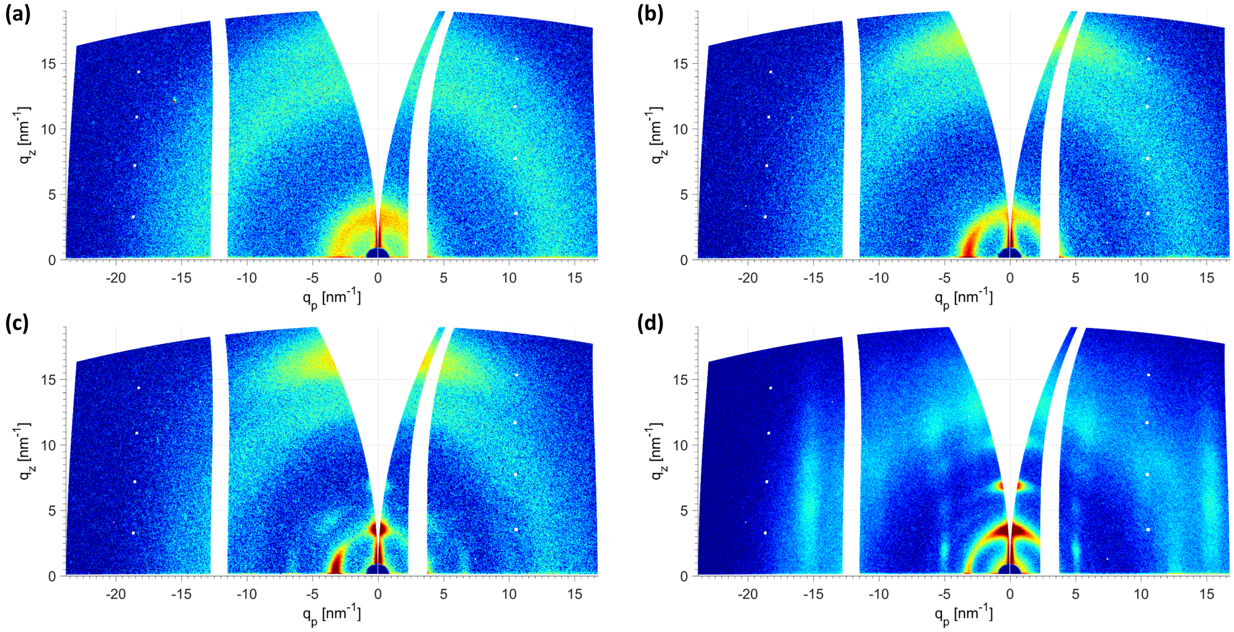

Figure S3: GIWAXS measurements of thin films of PDPP[Py]$_2$-T after different annealing programs. All measurements were performed at 20 °C at an angle of incidence of approximately 0.18°. a) Sample measured as-cast. b) Sample measured after annealing for 12 min at 180 °C. c) Sample measured after annealing for 12 min at 240 °C. d) Sample measured after annealing for 6 min at 289 °C and step-wise cooling.

Figs. S3(a-c) shows selected results from this series of measurements. The as-cast sample exhibits a broad and almost isotropic (1 0 0) reflection indicating poorly ordered crystals without preferred orientation (Fig.S3(a)). After annealing at 180 °C, the sample shows an

increase in face-on oriented crystals (Fig.S3(b)). Upon further increase of the annealing temperature an increase of edge-on oriented crystals can be observed, resulting in a binary distribution of orientations (Fig.S3(c)). Judging from the intensity of the reflections and the appearance of additional reflections, the overall order of the crystals increases with increasing annealing temperature. Fig. S3(d) shows for comparison the GIWAXS pattern of a new sample, which underwent the finally chosen annealing program. It is the same measurement as shown in Fig. 5(c). This mostly edge-on oriented thin film with well ordered crystals was obtained by annealing an as-cast film for 6 min at 289 °C followed by a step-wise cooling to 20 °C as sketched in Fig. S4 (a). The isothermal steps during cooling were inserted to increase the time available for ordering. The first isothermal step of 7 min was at a temperature of 259 °C, as PDPP[Py]$_2$-T showed the highest degree of ordering in temperature dependent powder WAXS measurements at this temperature during heating. Another isothermal step of 12 min was added at a temperature of 130 °C where PDPP[Py]$_2$-T showed the first signs of reorganization in temperature dependent powder WAXS measurements during heating.

To monitor the state of the sample during the final annealing procedure and to make sure that the sample did not melt, short GIWAXS measurements were performed during the isothermal steps of the temperature program. Notwithstanding the short exposure time the (1 0 0) reflection was clearly visible at all temperatures (cf. Fig. S4(b) and (c)), confirming that the sample did not melt even at the highest temperature of 289 °C, which is nominally above the melting temperature. The intensity of the reflections further increased upon cooling. The difference in melting temperature observed in DSC and GIWAXS is likely related to a small error in the temperature calibration of the GIWAXS sample stage.

To ensure comparability between the different PDPP samples, PDPP[T]$_2$-T and PDPP[T]$_2$-T$_{DEG}$ were subjected to an analogous thermal program with the difference that crystallization started from the molten state. PDPP[T]$_2$-T was heated with 30 K min^{-1} to 300 °C and kept there for 6 min. The sample was then cooled with 10 K min^{-1} to 20 °C interrupted by two isothermal steps (7 min at 260 °C and 12 min at 130 °C). PDPP[T]$_2$-T$_{DEG}$ was heated

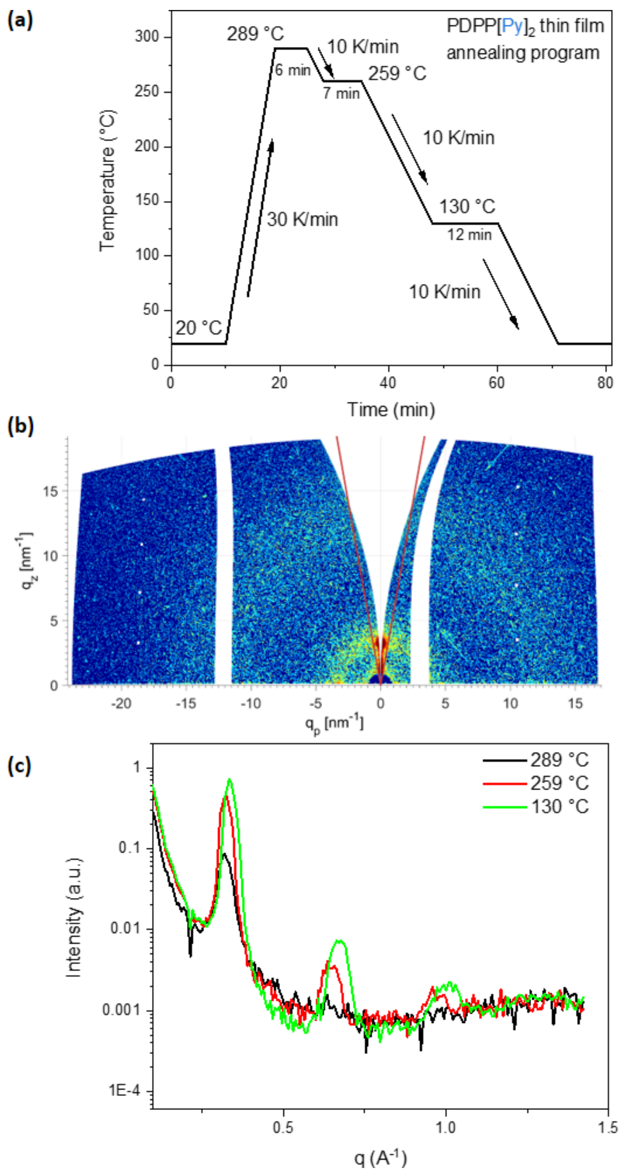

Figure S4: a) Annealing program of PDPP[Py]$_2$-T thin film samples. b) GIWAXS measurement at 289 °C at an angle of incidence of approximately 0.18°. c) Intensity line profiles extracted from GIWAXS measurements performed at different temperatures during cooling run of the annealing process. Red lines in b) indicate the integration range used to obtain the intensity line profiles shown in c).

with 30 K/min to 250 °C and kept there for 6 min. The sample was then cooled with 10 K/min to 20 °C interrupted by two isothermal steps (7 min at 200 °C and 12 min at 130 °C).

Thin Film WAXS Measurements in Reflection Geometry

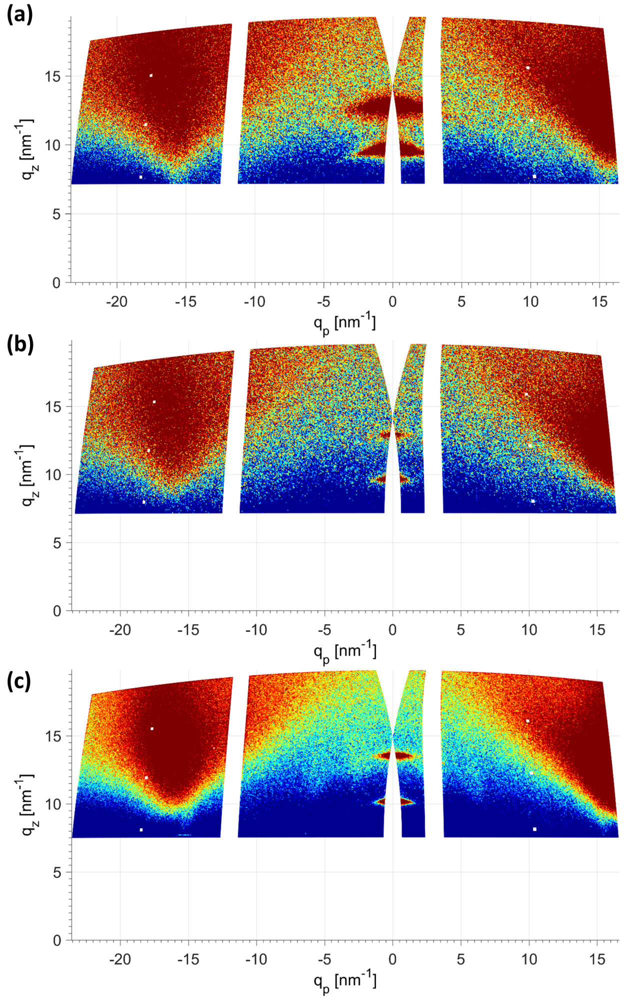

Figure S5: WAXS measurements on thin films in reflection geometry with an angle of incidence of 10° of a) PDPP[T]$_2$-T and b)PDPP[T]$_2$-T$_{DEG}$ after cooling from melt and c) PDPP[Py]$_2$-T after annealing at 289 °C. All measurements were performed at 20 °C.

To confirm the dominance of edge-on orientation in thin films after the appropriate sample treatment we performed WAXS measurements in reflection geometry with an incident angle of $\alpha_i \approx 10°$. The larger angle of incidence compared to the GIWAXS measurements allows to probe the reciprocal space at larger q_z values. The resulting scattering patterns are shown in Fig. S5. The data confirm the presence of (300) and (400) reflections on the q_z-axis. Additionally, the absence of a π-π-stacking peak is in keeping with the previous conclusion that there are little to no face-on oriented crystals. The lobes of high intensity visible on the

left and right side of the images originate from the silicon substrate.

Molecular Ordering in b-c Plane

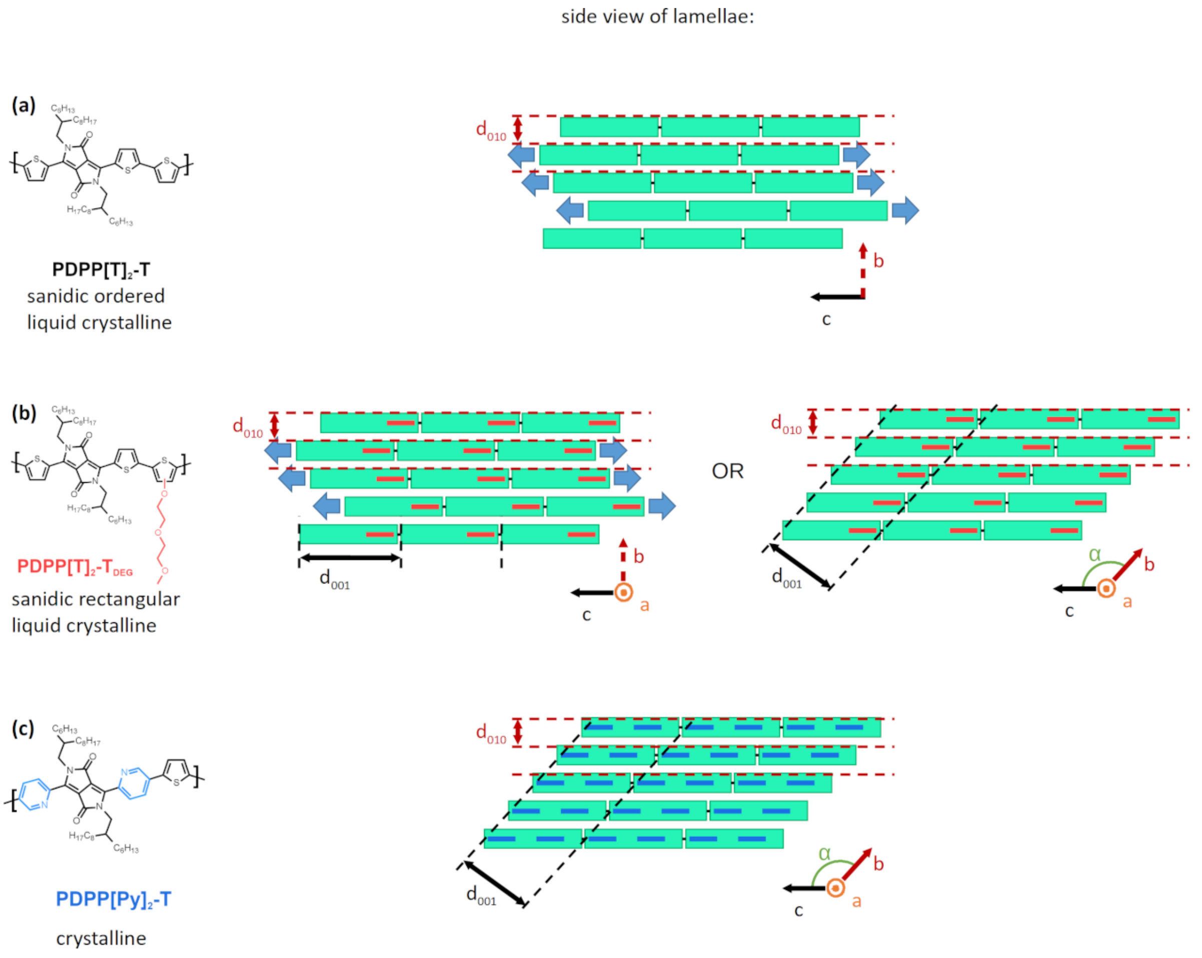

Figure S6: Sketches of suggested molecular arrangements in PDPP[T]$_2$-T (a), PDPP[T]$_2$-T$_{DEG}$ (b) and PDPP[Py]$_2$-T (c) in the b-c plane. In PDPP[T]$_2$-T neighboring stacked backbones are shifted randomly against one another in c-direction as indicated by the thick blue arrows. In PDPP[T]$_2$-T$_{DEG}$, based on the scattering results in Fig. 5(b), two scenarios are possible: neighboring stacked backbones are randomly shifted in c-direction (indicated by the blue arrows) (left); neighboring stacked backbones are registered in c-direction (right). In PDPP[Py]$_2$-T neighboring stacked backbones are registered in c-direction.

These sketches are complementary to Fig. 6 in the main text and illustrate the proposed molecular ordering of the three investigated PDPPs in b-c plane. Based on the scattering results in Fig. 5(b), we propose two scenarios for the molecular arrangement in PDPP[T]$_2$-T$_{DEG}$: 1) neighboring stacked backbones are randomly shifted in the c-direction, so that there is no registration between the b- and c-directions; 2) neighboring stacked backbones are registered in the c-direction. Although we cannot clearly distinguish between the two possible

arrangements, the second scenario, in which the stacks are registered in the c-direction, seems the most likely. Indeed, the absence of correlation between the polymer backbones in c-direction would imply a limited correlation length of the c-planes in b-direction. This in turn should result in a streak-like (0 0 1) reflection, which, in contrast, appears to be a well-defined sharp reflection as shown in Fig. 5(b) in the main text (peak 2 in Fig. 5(b)). Thus, we conclude that the second of the proposed scenarios is more consistent with the measured GIWAXS pattern in Fig. 5(b).

Positions of Bragg Reflections as Observed in WAXS and GIWAXS

Table S1: Peak positions of PDPP[T]$_2$-T determined from WAXS and GIWAXS measurements.

	WAXS	GIWAXS			Indexing
Peak No.	q[Å^{-1}]	q_p[Å^{-1}]	q_z [Å^{-1}]	$\|q\|$ [Å^{-1}]	$(h\ k\ l)$
1	0.322	0.000	0.339	0.339	1 0 0
2	0.647	0.000	0.673	0.673	2 0 0
3	0.972	0.000	1.006	1.006	3 0 0
4	$\approx$ 1.30	0.000	1.331	1.331	4 0 0
5	1.605	1.635	0.000	1.635	0 1 0

Table S2: Peak positions of PDPP[T]$_2$-T$_{DEG}$ determined from WAXS and GIWAXS measurements.

	WAXS	GIWAXS			Indexing
Peak No.	q[Å^{-1}]	q_p[Å^{-1}]	q_z [Å^{-1}]	$\|q\|$ [Å^{-1}]	$(h\ k\ l)$
1	0.332	0.000	0.336	0.336	1 0 0
2	-	0.322	0.173	0.366	0 0 1
3	$\approx$ 0.59	0.323	0.509	0.603	1 0 1
4	0.664	0.000	0.669	0.669	2 0 0
5	0.992	0.000	1.000	1.000	3 0 0
6	1.698	1.717	0.000	1.717	0 1 0

Table S3: Peak positions of PDPP[Py]$_2$-T determined from WAXS and GIWAXS measurements and peak positions calculated from the suggested unit cell: $a = 20.17\,\text{Å}^{-1}$, $b = 5.46\,\text{Å}^{-1}$, $c = 16.82\,\text{Å}^{-1}$ $\alpha \approx 131.4°$, $\beta \approx 100.0°$, $\gamma \approx 97.0°$.

	WAXS	GIWAXS			Indexing	calculated		
Peak No.	q[Å^{-1}]	q_p[Å^{-1}]	q_z [Å^{-1}]	$\lvert q\rvert$ [Å^{-1}]	$(h\ k\ l)$	q_p[Å^{-1}]	q_z [Å^{-1}]	$\lvert q\rvert$ [Å^{-1}]
1	0.334	0.000	0.342	0.342	1 0 0	0.000	0.334	0.334
2	0.530	0.502	0.183	0.534	0 0 1	0.498	0.181	0.530
3	0.669	0.000	0.677	0.677	2 0 0	0.000	0.668	0.668
4	-	0.501	0.516	0.719	1 0 1	0.498	0.515	0.717
5	-	0.539	0.800	0.965	2 0 1	0.498	0.849	0.985
6	1.000	0.000	1.023	1.023	3 0 0	0.000	1.002	1.002
7	1.164	-	-	-	1 $\bar{1}$ 2	1.151	0.177	1.165
8	1.267	1.255	0.000	1.255	0 $\bar{1}$ 3	1.247	0.024	1.247
9	-	0.573	1.131	1.268	-	-	-	-
10	-	0.250	1.277	1.301	-	-	-	-
11	1.337	0.000	1.354	1.354	4 0 0	0.000	1.336	1.336
12	1.555	1.544	0.162	1.552	$\bar{1}$ 1 0	1.534	0.185	1.546
13	1.620	1.542	0.522	1.628	0 1 0	1.534	0.519	1.620
14	1.732	1.534	0.852	1.755	1 1 0	1.534	0.853	1.756

Determination of the Reciprocal Unit Cell of PDPP[Py]$_2$-T

The reconstruction of the reciprocal unit cell of a crystal based on the information obtained from GIWAXS measurements on polycrystalline samples with uniaxial orientation distribution is not straightforward. Due to the rotational symmetry of the intensity distribution in reciprocal space around the alignment direction, the position of the reciprocal lattice points can be readily described in the form of cylindrical coordinates q_p, q_z and ϕ. While the radial distance q_p and the height q_z of a specific reciprocal lattice point can be directly read off from the measurement, the azimuthal angle ϕ can not be directly obtained as a result of the rotational symmetry.

In consequence for the reconstruction of the reciprocal unit cell, even if one observes the (1 0 0), (0 1 0), and (0 0 1) reflections (or higher orders of them) directly, only the length of the reciprocal unit cell vectors a^*, b^* and c^* can be obtained, while the angles between them remain unknown. If, as it is often the case for board-shaped polymers, one of these three reflections lies directly on the q_z-axis, it is additionally possible to extract the two angles between the reciprocal unit cell vector corresponding to the reflection on the q_z-axis and the other two reciprocal unit cell vectors from the measurement. For example, if it is the (1 0 0) reflection that lies on the q_z-axis, then one can directly read off β^* and γ^*. The third angle α^* can be determined from the position of a reflection with mixed indices, with $k \neq 0$ and $l \neq 0$. The radial distance $G_{p,hkl}$ of the reciprocal lattice vector $\vec{G}_{hkl}$ corresponding to such a reflection is a function of the last unknown angle, which can therefore be determined, if all other parameters of the reciprocal unit cell are known.

In the following, the reciprocal lattice vectors $\vec{G}_{hkl}$ are described in terms of cylindrical coordinates, as mentioned above. The z-axis of the coordinate system is oriented parallel to the normal of the substrate. The radial length $G_{p,hkl}$ and the height $G_{z,hkl}$ of a reciprocal lattice vector can be directly obtained from the q_p and the q_z position of an $(h\ k\ l)$ reflection as observed in GIWAXS. In the GIWAXS measurements of the PDPP[Py]$_2$-T sample the (1 0 0), (0 1 0) and (0 0 1) reflections can be observed. Therefore, the length of the reciprocal

unit cell vectors a^*, b^* and c^* can be obtained from the GIWAXS data. The (1 0 0) reflection (and higher orders of it) lie directly on the q_z-axis corresponding to an edge-on orientation. Furthermore, the angle β^* , between $\vec{a}^*$ and $\vec{c}^*$ and the angle γ^* between $\vec{a}^*$ and $\vec{b}^*$ are given by $\beta^* = \arctan(G_{p,001}/G_{z,001})$ and $\gamma^* = \arctan(G_{p,010}/G_{z,010})$.

To determine the last unknown angle of the reciprocal unit cell α^*, it is necessary to determine the azimuthal angles ϕ_{0k0} and ϕ_{00l} of the reciprocal lattice vectors $\vec{G}_{0k0}$ and $\vec{G}_{00l}$ as will become obvious from eq. (4). Without further restriction ϕ_{0k0} can be set to 0 by choosing a coordinate system with a suitable orientation. As mentioned, to determine ϕ_{00l} a reflection with mixed indices with $k \neq 0$ and $l \neq 0$ is necessary. The lattice vector $\vec{G}_{hkl}$ corresponding to such a reflection is defined by:

$$\vec{G}_{hkl} = h \cdot \vec{G}_{100} + k \cdot \vec{G}_{010} + l \cdot \vec{G}_{001} \tag{1}$$

As this equation also holds for $\vec{G}_{p,hkl}$, the projection of $\vec{G}_{hkl}$ onto the q_x-q_y-plane is:

$$\vec{G}_{p,hkl} = \vec{G}_{p,0k0} + \vec{G}_{p,00l}. \tag{2}$$

Since the reciprocal lattice vector $\vec{G}_{100}$ is parallel to the q_z-axis, $\vec{G}_{p,100}$ is equal to zero and does not contribute. As sketched in Fig. S7, the vector $\vec{G}_{p,hkl}$ has to fulfill two conditions. On the one hand, it has to have the length $|\vec{G}_{p,hkl}|$ determined from the GIWAXS measurement illustrated by the green dashed circle in Fig. S7. On the other hand, as it also has to fulfill equation 2, it has to end on a circle with radius $|\vec{G}_{p,00l}|$ around the tip of $\vec{G}_{p,0k0}$ illustrated by the blue dashed circle in Fig. S7. Both conditions are fulfilled at the intersections of the two circles. Both intersections represent valid solutions, where one results in the same unit cell as the other except for being mirrored at the q_x-q_z-plane. The intersections define the orientation of $\vec{G}_{p,00l}$ and $\vec{G}_{p,hkl}$, therefore allowing to determine the azimuthal angle ϕ_{00l}

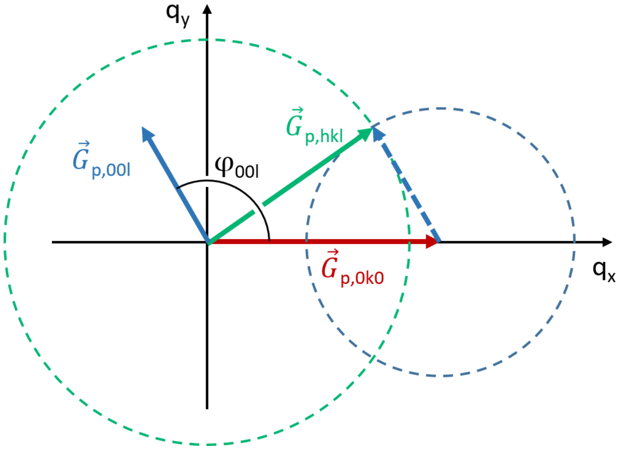

Figure S7: Sketch of the q_x-q_y-plane in reciprocal space with the projections of the $\vec{G}_{0k0}$, $\vec{G}_{00l}$ and $\vec{G}_{hkl}$ vectors onto the plane. As $\vec{G}_{h00}$ is parallel to the q_z axis the direction of the $\vec{G}_{p,hkl}$ is solely given by the vectorial addition of $\vec{G}_{p,0k0}$ and $\vec{G}_{p,00l}$. The vector $\vec{G}_{p,hkl}$ has to lie on the green circle with the radius of $|\vec{G}_{p,hkl}|$ determined from GIWAXS measurements. Additionally $\vec{G}_{p,hkl}$ has to lie on the blue circle with radius $|\vec{G}_{p,00l}|$ (also determined from GIWAXS) around the tip of $\vec{G}_{p,0k0}$. The intersect of both circles defines the directions of $\vec{G}_{p,hkl}$ and $\vec{G}_{p,00l}$. Therefore, if $|\vec{G}_{p,0k0}|$, $|\vec{G}_{p,00l}|$ and $|\vec{G}_{p,hkl}|$ are known, one can determine the azimuthal angle ϕ_{00l} of the reciprocal lattice vector $\vec{G}_{00l}$.

using the law of cosines:

$$\phi_{00l} = 180° - \arccos\left(\frac{|\vec{G}_{p,0k0}|^2 + |\vec{G}_{p,00l}|^2 - |\vec{G}_{p,hkl}|^2}{2 \cdot |\vec{G}_{p,0k0}| \cdot |\vec{G}_{p,00l}|}\right) \tag{3}$$

The angle α^* of the reciprocal unit cell is then obtained through the dot product of $\vec{G}_{010}$ and $\vec{G}_{001}$.

$$\alpha^* = \arccos\left(\frac{\vec{G}_{0k0} \cdot \vec{G}_{00l}}{|\vec{G}_{0k0}| \cdot |\vec{G}_{00l}|}\right) \tag{4}$$

$$= \arccos\left(\frac{1}{|\vec{G}_{0k0}| \cdot |\vec{G}_{00l}|}\begin{pmatrix} |\vec{G}_{p,00l}|\cos(\phi_{00l}) \\ |\vec{G}_{p,00l}|\sin(\phi_{00l}) \\ G_{z,00l} \end{pmatrix} \cdot \begin{pmatrix} |\vec{G}_{p,0k0}| \\ 0 \\ G_{z,0k0} \end{pmatrix}\right) \tag{5}$$

$$= \arccos\left(\frac{|\vec{G}_{p,0k0}|\cos(\phi_{00l}) \cdot |\vec{G}_{p,00l}| + G_{z,00l} \cdot G_{z,0k0}}{|\vec{G}_{0k0}| \cdot |\vec{G}_{00l}|}\right) \tag{6}$$

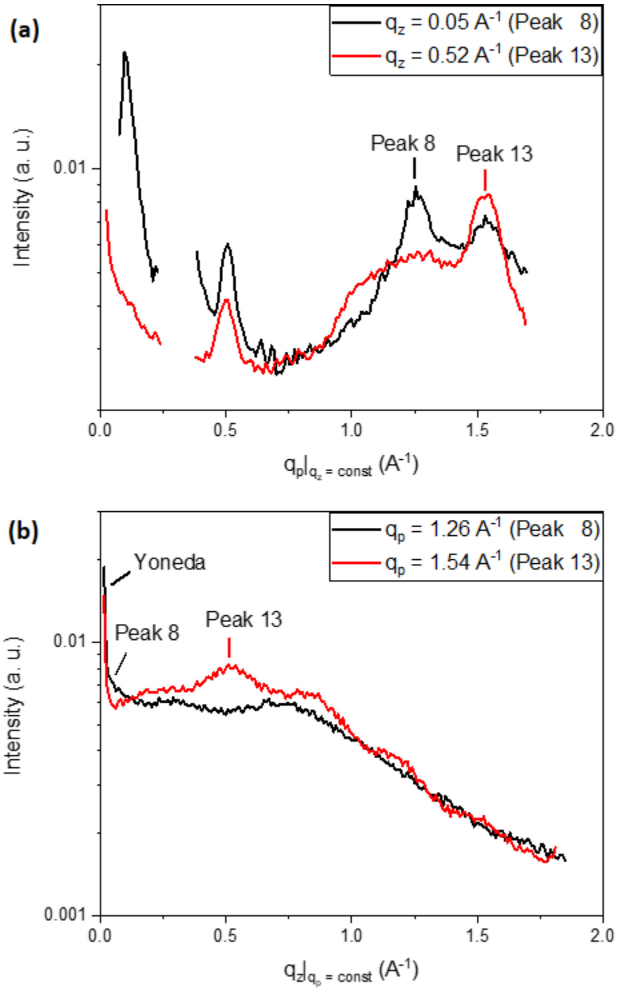

Figure S8: Intensity line profiles in (a) q_p and (b) q_z direction extracted from the GIWAXS measurement of PDPP[Py]$_2$-T shown in Fig. 5c. The width of the integration area was $0.1\,\text{Å}^{-1}$.

We now focus on the indexation of peak 8 observed in the GIWAXS measurement of PDPP[Py]$_2$-T. Although not well visible in the 2D-GIWAXS measurement (Fig. 5c), the existence of peak 8 is confirmed when looking at line profiles. Fig. S8 shows line profiles in q_z- and q_p-direction extracted from the 2D-GIWAXS measurement in Fig. 5 c) at the position of peak 8 and peak 13 in comparison. From the line profile in q_p-direction shown in Fig. S8 a) one can clearly determine the q_p position of peak 8. The q_z line profile of peak 8, shown in Fig. S8 b, clearly shows increased intensity around $q_z \approx 0.05\,\text{Å}^{-1}$ that does not solely originate from the Yoneda effect, as the comparison with the q_z line profile of peak 13 shows, where the intensity decreases much quicker with increasing q_z. Because of the increased intensity at small q_z values due to the Yoneda effect that overlaps with the scattered intensity of peak 8, the q_z position of peak 8 can not be clearly determined and only a certain q_z-range can be given. The q_z position of peak 8 is in the range of $q_z \approx 0.0\,\text{Å}^{-1}$ to $0.05\,\text{Å}^{-1}$.

To identify the correct indices of peak 8 the Miller indices h, k and l were all iterated from -5 to 5. All combinations that did not yield a reciprocal lattice vector $\vec{G}_{hkl}$ with a height of $q_z < 0.05\,\text{Å}^{-1}$ were discarded. For some of the remaining combinations there is no solution for α^*, due to geometric reasons. For example, if $\vec{G}_{p,0k0}$ is longer than $\vec{G}_{p,hkl}$ and $\vec{G}_{p,00l}$ is shorter than $|\vec{G}_{p,0k0}| - |\vec{G}_{p,hkl}|$, the circles in Fig. S7 do not intersect. In total this resulted in five possible hkl-combinations and therefore five different possible values for α^*. To determine the most likely unit cell, we checked if peak 7 ($q = 1.164\,\text{Å}^{-1}$) from the WAXS pattern could be indexed, and its expected q-position was calculated based on the five unit cells. Table S4 summarizes the results. The best fit for peak 7 was obtained with $\alpha^* = 45.7°$ resulting from indexing peak 8 as $(0\ \bar{1}\ 3)$.

Since the peaks in the GIWAXS measurement are rather broad and the accuracy to which their q-position can be determined is limited, only the angles of the reciprocal unit cell α^*, β^*, and $\gamma*$ determined from the GIWAXS measurements were used to calculate the final unit cell. The reciprocal lengths a^*, b^*, and c^* where instead determined from the (1 0 0),

Table S4: Possible Miller indices of Peak 8 observed in GIWAXS measurements of PDPP[Py]$_2$-T

Peak 8 Indexing	resulting	Peak 7 Indexing	Peak 7 calculated
$(h\ k\ l)$	$\alpha^*[°]$	$(h\ k\ l)$	$\|q\|[Å^{-1}]$
$\bar{3}$ 1 3	118.6	3 $\bar{1}$ $\bar{2}$	1.159
$\bar{2}$ 1 1	119.8	$\bar{2}$ 1 2	1.145
$\bar{1}$ 1 $\bar{1}$	44.2	3 0 $\bar{2}$	1.185
0 $\bar{1}$ 3	45.7	1 $\bar{1}$ 2	1.165
$\bar{1}$ 2 $\bar{4}$	14.0	0 $\bar{1}$ 5	1.147

(0 1 0), and (0 0 1) peaks in the WAXS pattern. This procedure results in the unit cell proposed in Table 2.
